# Letter from Europe

**Published:** 1857-04

**Authors:** Isidor Gluck

**Affiliations:** Vienna


*Letter from Europe.*—The following letter, from a former contributor to our pages, to the American Medical Monthly, possesses sufficient interest to warrant our transfer of it to the Journal:

VIENNA, January 20, 1857.

To the Editors of the American Medical Monthly:

I find myself again among old friends, well known abroad,—authorities on various subjects, whose opinions are so reliable that their endorsement is necessary before our transatlantic friends dare to consider discoveries and truths as such. It is strange that after a period of eight years, I should find all faces unchanged, and as to their expression quite the same.

You may still observe Rokitansky searching among the dead with his wonted countenance,—showing the silent but nevertheless deep thinker,—in order to confirm long established truths, discovered and repeatedly tested, silent as he is, by himself. One has to catch the words which apparently fall by chance from his lips, yet however rare they may be, it well repays the trouble, for every word he utters, though it gives but a hint as to the morbid appearance, is sufficiently instructive to reward one for attentively watching for his remarks.

Intently looking through his eye-glass, both hands kept in his pockets, he now and then raises the one to point to a morbid condition, overlooked or not sufficiently considered, lifting at the same time his head turned toward the student, a smile on his lips serving as words—for to speak is too much trouble to one who, like Rokitansky, is busy thinking ahead. Now and then a remark for further explanation is all that joins the smile, and still it is sufficient to explain what may and might be seen. What else should he do? On the positive field of activity he moves, stories are not needed to fill up the time and amuse the serious student.

The wards I often visited eight years ago, I strolled through once more. It is a division under Prof. Sigmund's direction. This man alters neither in manner nor in speech. Courteous in both, he is loud in voice to pupil and patient, of course in order to be well understood, for students and doctors, coming from all parts of the world, need a clear and distinct explanation, given as it is in the German language, not well understood by foreigners. You may hear quite as often English or French remarks made for the benefit of those who know neither German nor Latin, which latter, being a Hungarian, he speaks by predilection.

Seated on a low window, Prof. Sigmund is surrounded by native and foreign students and physicians, some of whom are eagerly glancing at the voluptuous forms of the half naked women, introduced by the nurse, one by one, out of the soldier-like formed line, who mentions the name of the patient and the number of her bed, while the assistant surgeon (Secundarius) proclaims the diagnosis and treatment adopted. The patients, most of whom are emaciated and haggard, yet now and then well fed (if long inmates of the hospital), of all ages from 14 to 60 and upwards, approach with spoon in hand, half naked, shirt and jacket being turned down, each one performing three well drilled movements.

1st. Appearing in front with head erect.

2d. Turning around and showing on her neck and back the different erythemata, pustulæ, or any form you choose, of the various syphylides.

3d. Turning again in front and opening the mouth for inspection, reaching forward at the same time the spoon for depressing the tongue to the Professor. I must confess the spatula of Dr. Horace Green is better calculated for such a purpose, and I have suggested the idea of giving to the upper end of the spoon of these patients the shape of Green's spatula.

Thus drilled, the patients appear daily, from 100 to 200 in number. They are supposed to talk German, but a polyglotist has great difficulty in making it out or in finding any similarity to any language, for one talks

German which sounds like Bohemian, and another talks Bohemian with such a German accent that it sounds neither German nor Bohemian, so that German and Bohemian students look at each other inquiringly; Professor, nurse, and student, are left to guesswork, luckily helped along by the loudly speaking syphilitic forms, well marked, and telling.

In front of the window is a stand, about five feet high, ascended on its side by a ladder, which serves as a bed to the female patient, who reclines on it so as to be fully exposed while being examined, without giving the trouble of stooping, or the inconvenience of remaining in that position, an improvement well worth imitating, for the parts being almost in a direct line with the eye of the spectator, the latter does not become fatigued, for the reason that he can stand erect all the time.

The applications of caustic, or, as frequently used here, of arsenic solutions, are made by the secundarius, and the inunctions with mercurial ointments, according to Sigmund's most recent practice, are made by the nurses, and not left to the patients. The patient kept on the stand is laid comfortably, and exhibits without fatigue, as long as needed. The French mode of examining by means of a sheet, is not used in this ward, for the simple reason that the exhibitors would sooner blush when covered by a white cloth than they would without it, coming as they do from various quarters, none so pure as to need a veil.

Sigmund's contributions to syphilidology are well worth being studied, and I shall take another opportunity of speaking of his treatise on Radesyge,* for the purpose of examining which disease he intends to travel into Spain, having already confronted it, and shown its similarity in various distant countries, although appearing under the most different names. Not less interesting is his contribution on "Sleeplessness in Chronic Syphilis," periodically returning at certain hours, which he made to the *Œsterreichische Zeitschrift, für Practische Heilkunde*, published by the Doctors College of the Medical Faculty in Vienna, edited by Dr. J. Knolz, and assisted by Dr. G. Preyes, an eminent man in many respects, who has the care and entire management of it. The journal, although now only three years old, ranks among the first in the country, having original communications of great interest from the highest authorities. Its last volume, consisting of fifty-two numbers, (one appearing every week), contains, among other articles by my friend Dr. C. Brann, Professor of Obstetrics, formerly of Trent, now of Vi-

---

* This is a name given in Norway to a disease analogous to the Yaws. It is by some reckoned as a variety of lepra.—ED. AMER. MED. MONTHLY.

enna, a valuable article on the "Contraction or Motility of the Uterus, produced by Carbonic Acid Gas." Professor Dietl, of Cracow, added through its columns to the etiology of Diabetes Mellitus. Haller, of Vienna, published in this journal his observations on the *Typhus Laryngo-Ulcer.* Heyfelder, Nusser, and Pabrubein, each of them, as well as Professors Dumreicher and Schuh, have contributed highly scientific articles, which richly deserve translation. Dr. Preyes, himself, besides editing the journal, has contributed largely, not only reviewing in a just and scientific way literary works, but also by reporting the transactions of societies. In short, the *Œsterreichische Zeitschrift* is a rising and promising journal, ably conducted by its highly educated coëditor, who spares no trouble nor expense in placing it upon an equal footing with the *Medical Zeitschrift.*

On my proposed return home (to America), I passed last December in Dresden and Leipsic, thence going to Berlin. I spent a pleasant morning at Ruete's clinique for diseases of the eye, in Leipsic. My courteous friend, Coccius (who by the way promised me contributions for the American Medical Monthly) gave me the opportunity of visiting the Institution for the relief of the Blind, erected in a fine neighborhood, enjoying fresh air, and light, and situated almost in the midst of a garden, created many years ago through the influence and care of Professor Ritterich, who, strange to say, though old, pays a just tribute to new inventions, and warmly applauds the results gained by means of the eye speculum. No prejudice whatsoever keeps him from examining, pondering, and judging for himself. The gray Professor, affable and kind in his manner and conversation, is still seated over the folio volumes, although his sight is comparatively unequal to the work his mind intends to carry out. Professor Ritterich has contributed much to German ophthalmic surgery, and was an excellent operator.

The editor of *Schmidt's Jahrbücher* I found in the fourth story. Professor Richter is a tall, thin, slender man, who talks obligingly. Arrived at his door after a laborious ascension, I said to Coccius, who accompanied me, that he might get spiritual contributions if he needed any, at first hand, being so near the sky, but luckily he makes compilations only, and uses the spirit of others.

The last visit, as usual, I paid to the post office, and finding there letters from relatives and friends begging me to return, it needed only my mother's, received at the same time, desiring me in a few words to come back again, to make me so completely *homesick*, that I returned immediately, and now find myself settled in Vienna.

To my numerous personal and professional transatlantic friends, I take this means of saying, "Good Bye," and of assuring them of my regards for their kind consideration toward me during my residence in America. As a representative of the American Medical Association, at the Congress of Physicians and Naturalists, in Vienna, I will send in my report at the proper occasion. As a member of the various medical and literary societies in New York, as well as in America in general, I beg leave to state that I shall always be happy to act, if needed, in their interest, upon receiving instructions for so doing.

My personal and literary connection with you, the remembrance of which will always be dear to me, shall be unchanged, though at a distance.

I remain near "my sweet home," yet with the highest consideration and regards for you. Yours truly,

ISIDOR GLUCK, M. D.

